# Association between ZFHX3 and PRRX1 Polymorphisms and Atrial Fibrillation Susceptibility from Meta-Analysis

**DOI:** 10.1155/2021/9423576

**Published:** 2021-12-14

**Authors:** Liting Wu, Min Chu, Wenfang Zhuang

**Affiliations:** Medical Laboratory, Shidong Hospital, Affiliated to University of Shanghai for Science and Technology, No. 999, Shiguang Road 200438, Yangpu District, Shanghai, China

## Abstract

**Background:**

Atrial fibrillation (AF) is a common, sustained cardiac arrhythmia. Recent studies have reported an association between ZFHX3/PRRX1 polymorphisms and AF. In this study, a meta-analysis was conducted to confirm these associations. *Objective and Methods*. The PubMed, Embase, and Wanfang databases were searched, covering all publications before July 20, 2020.

**Results:**

Overall, seven articles including 3,674 cases and 8,990 healthy controls for ZFHX3 rs2106261 and 1045 cases and 1407 controls for PRRX1 rs3903239 were included. The odds ratio (OR) (95% confidence interval (CI)) was used to assess the associations. Publication bias was calculated using Egger's and Begg's tests. We found that the ZFHX3 rs2106261 polymorphism increased AF risk in Asians (for example, allelic contrast: OR [95% CI]: 1.39 [1.31–1.47], *P* < 0.001). Similarly, strong associations were detected through stratified analysis using source of control and genotype methods (for example, allelic contrast: OR [95% CI]: 1.51 [1.38–1.64], *P* < 0.001 for HB; OR [95% CI]: 1.31 [1.21–1.41], *P* < 0.001 for PB; OR [95% CI]: 1.55 [1.33–1.80], *P* < 0.001 for TaqMan; and OR [95% CI]: 1.31 [1.21–1.41], *P* < 0.001 for high-resolution melt). In contrast, an inverse relationship was observed between the PRRX1 rs3903239 polymorphism and AF risk (C-allele *vs.* T-allele: OR [95% CI]: 0.83 [0.77–0.99], *P*=0.036; CT *vs.* TT: OR [95% CI]: 0.79 [0.67–0.94], *P*=0.006). No obvious evidence of publication bias was observed.

**Conclusions:**

In summary, our study suggests that the ZFHX3 rs2106261 and PRRX1 rs3903239 polymorphisms are associated with AF risk, and larger case-controls must be carried out to confirm the abovementioned conclusions.

## 1. Introduction

Atrial fibrillation (AF) is a common form of arrhythmia, with an incidence of approximately 1% among adults worldwide [1, 2]. Previous studies have demonstrated that AF significantly increases the social and economic burden in both developed and developing countries [3]. Additionally, AF is the main cause of heart failure and stroke [4, 5]. A variety of structural heart diseases and systemic diseases are related to AF, including congestive heart failure, cardiomyopathy, pulmonary heart disease, essential hypertension, and hyperthyroidism [6, 7], while age, obesity, smoking, excessive drinking, and drug use also contribute to the development of AF [6, 8]. Thus far, the exact pathogenesis of AF remains unclear. However, many studies have suggested that genetic factors play an important role in AF occurrence and development [9]. In fact, common genetic variants (a multitude of single-nucleotide polymorphisms (SNPs)) associated with AF have been detected in genome-wide association studies (GWASs) [10–12], such as endothelial nitric oxide synthase 786 T/C, CYP11B2 rs1799998, KCNE1 G38S, and caveolin-1 rs3807989 [9, 13–15].

Two independent GWASs identified significant associations between rs2106261 and rs7193343 polymorphisms in the zinc finger homeobox 3 (ZFHX3) gene and AF susceptibility in various populations of European ancestry [16, 17]. ZFHX3 is located on chromosome 16q22. Benjamin et al. [16] indicated that the rs2106261 SNP in ZFHX3 was associated with AF (OR = 1.19; *P*=2.76 × 10^−7^). At the same time, Gudbjartsson et al. [17] assessed another SNP (rs7193343) in ZFHX3, which was confirmed to be related to AF in Icelandic individuals (OR = 1.21, *P*=1.4 × 10^−10^).

Paired homeobox 1 (PRRX1) encodes a homeodomain transcription factor that is highly expressed in the developing heart [18]. Fetal lung vascular development was impaired in a PRRX1 knockout mouse model [19]. The expression pattern of PRRX1 in the mouse atria was evaluated; both genes were overexpressed in the left atrium when compared to the right atrium [20]. These results suggest that PRRX1 may play a vital role in heart diseases, including AF. In a subsequent meta-GWAS, the PRRX1 rs3903239 variant was associated with AF risk (*P*=8.4 × 10^−14^) [21].

Taking into consideration the more precise assessment of the ZFHX3 rs2106261 and PRRX1 rs3903239 variants in AF risk, we must first perform a meta-analysis of all eligible case-control studies to confirm the associations [18, 22–27].

## 2. Materials and Methods

### 2.1. Identification and Eligibility of Relevant Studies

The PubMed, Embase, and Wanfang databases were selected. The last search was conducted on July 20, 2020, with the search terms including the keywords “ZFHX3” or “zinc finger homeobox 3,” “PRRX1” or “paired related homeobox 1,” “polymorphism” or “variant,” and “atrial fibrillation.” After the abovementioned search, a total of 96 publications were identified, of which 7 met the inclusion criteria.

### 2.2. Criteria for Inclusion and Exclusion

The studies included in the analysis met all of the following conditions: (a) the study assessed the correlation between AF and the ZFHX3 rs2106261 polymorphism and/or PRRX1 rs3903239 polymorphism; (b) unpaired case-control studies; and (c) sufficient genotypes in cases and controls. In addition, the following exclusion criteria were applied: (a) no control group; (b) no genotype frequency was available; and (c) previous publications were repeated.

### 2.3. Data Extraction

Two of the authors extracted all data independently and complied with the selection criteria. The following items were collected: author's name, ethnicity, year of publication, total of each genotype case/control number, country, source of control, genotyping methods, and Hardy–Weinberg equilibrium (HWE) of controls.

### 2.4. Quality Score Assessment

The Newcastle–Ottawa Scale (NOS) was used to assess the quality of each study and evaluate all aspects of the methodology, including case selection, comparability between groups, and exposure determination. The NOS has a total score of 0–9 stars. Research with a score greater than 7 is considered a high-quality study [28].

### 2.5. Statistical Analysis

Based on the genotype frequencies of the cases and controls, the probability odds ratio (OR) with 95% confidence interval (CI) was used to measure the strength of the association between the polymorphisms and AF. First, we conducted a subgroup analysis stratified by race. The source of the control subgroup analysis was carried out in two categories: population based (PB) and hospital based (HB).

The statistical significance of the OR was determined using the *Z*-test. The fixed and random effect models were used to calculate the combined OR. The *Q*-test (*P* ≥ 0.10) indicated heterogeneity between the included studies. If significant heterogeneity was detected, the random-effects model (DerSimonian–Laird method) was used, but otherwise, the fixed-effects model (Mantel–Haenszel method) was selected [29, 30]. For ZFHX3 rs2106261, we investigated the relationship between genetic variants and AF risk in allelic contrast (A-allele *vs.* G-allele), homozygote comparison (AA *vs.* GG), the dominant genetic model (AA + AG *vs.* GG), heterozygote comparison (AG *vs.* GG), and recessive genetic models (AA *vs.* AG + GG). For PRRX1 rs3903239, C-allele vs. T-allele, CT *vs.* TT, CC *vs.* TT, CC + CT *vs.* TT, and CC *vs.* CT + TT models were applied. Funnel plot asymmetry was assessed using Begg's test, and publication bias was assessed using Egger's test [31]. The departure of frequencies from expectation under HWE was assessed using the *χ*^2^ test in the controls through the Pearson chi-square test (*P* < 0.05 was considered significant) [32]. All statistical tests for this meta-analysis were performed using Stata software (version 11.0; StataCorp LP, College Station, TX, USA).

### 2.6. ZFHX3 and PRRX1 Interaction Networks

To fully understand the role and potential functional partners of ZFHX3 and PRRX1 in AF, the String online server (https://string-db.org/) was used to create a gene-gene interaction network of ZFHX3 and PRRX1 [33].

## 3. Results

### 3.1. Eligible Studies

**Figure 1:**
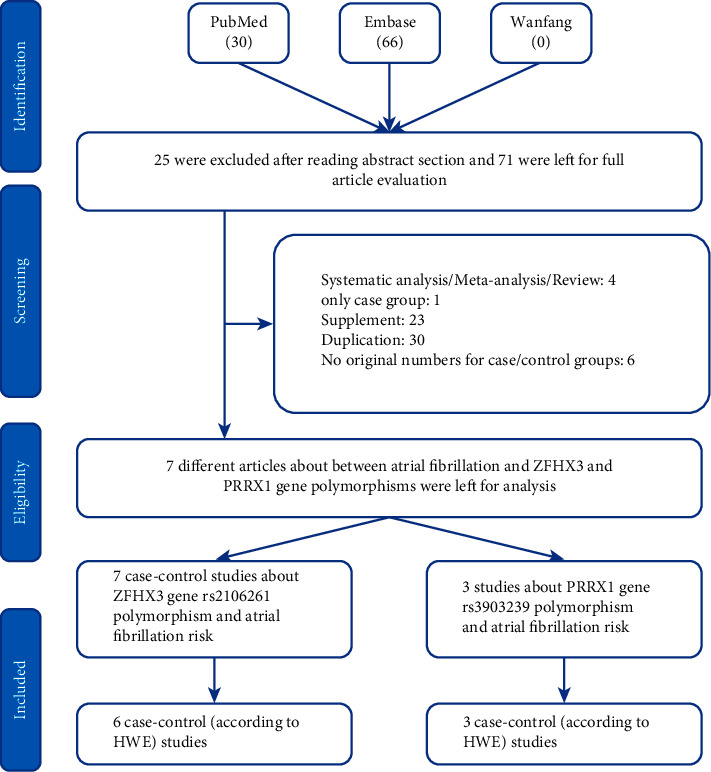
A flowchart showing the search strategy applied to search the related papers for ZFHX3 rs2106261 and PRRX1 rs3903239 polymorphisms and AF risk.

**Figure 2:**
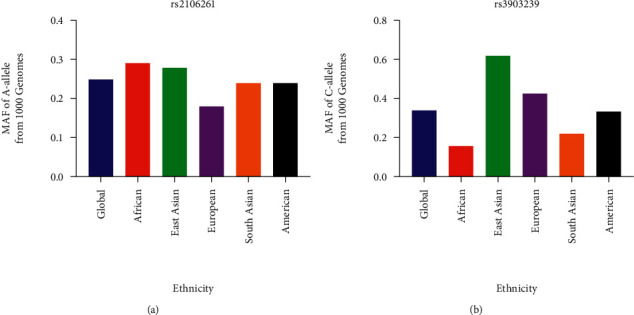
MAF for the gene polymorphisms among different ethnicities. Vertical line, MAF; horizontal line, ethnicity type. EAS: East Asian; EUR: European; AFR: African; AMR: American; and SAS: South Asian. (a) rs2106261 and (b) rs3903239.

In total, 96 articles were collected from the PubMed, Embase, and Wanfang databases. Of these, 89 articles were excluded (25 unrelated articles, 4 systematic/meta-analysis studies, 1 with only a case group, 23 supplements, 30 duplications, and 6 with no original numbers for case/control groups) (Figure 1). Finally, seven articles were identified in the current analysis, including 3,674 cases and 8,990 healthy controls related to the ZFHX3 rs2106261 polymorphism and 1045 cases and 1407 controls for the PRRX1 rs3903239 polymorphism. The characteristics of each study are presented in Table 1. In addition, the minor allele frequency (MAF) reported from the five main worldwide populations in the 1000 Genomes Browser were checked (https://www.ncbi.nlm.nih.gov/snp/): African, European, East Asian, American, and South Asian populations (Figure 2); the MAF was similar to the average level in our current case and control groups.

### 3.2. ZFHX3 rs2106261 and AF Risk

**Figure 3:**
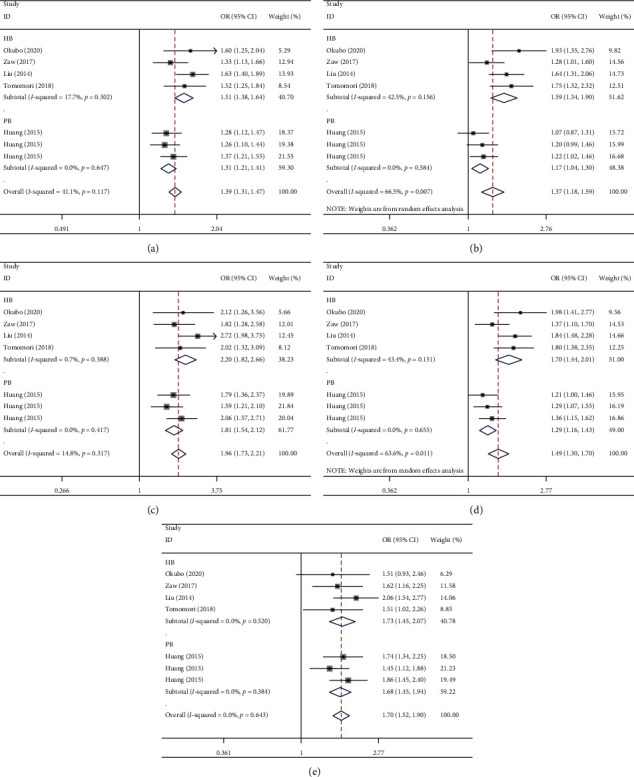
Forest plot of AF risk associated with ZFHX3 rs2106261 polymorphism in all genetic models by source of the control subgroup. The squares and horizontal lines correspond to the study-specific OR and 95% CI. The area of the squares reflects the weight (inverse of the variance). The diamond represents the summary OR and 95% CI. (a) A-allele vs. C-allele; (b) AC vs. CC; (c) AA vs. CC; (d) AA + AC vs. CC; and (e) AA vs. AC + CC.

In the overall analysis, increased associations were observed in five genetic models in Asians: allelic contrast (OR [95% CI] = 1.39 [1.31–1.47], *P*_heterogeneity_ = 0.117, *P* < 0.001, Figure 3(a)), heterozygote comparison (OR [95% CI] = 1.37 [1.18–1.59], *P*_heterogeneity_ = 0.007, *P* < 0.001, Figure 3(b)), AA *vs.* CC (OR [95% CI] = 1.96 [1.73–2.21], *P*_heterogeneity_ = 0.317, *P* < 0.001, Figure 3(c)), the dominant model (OR [95% CI] = 1.49 [1.30–1.70], *P*_heterogeneity_ = 0.011, *P* < 0.001, Figure 3(d)), and AA *vs.* AC + CC (OR [95% CI] = 1.70 [1.52–1.90], *P*_heterogeneity_ = 0.643, *P* < 0.001, Figure 3(e)) (Table 2).

In the subgroup analysis by source of control, the ZFHX3 rs2106261 A-allele or AA genotype acted as a risk factor in both HB and PB subgroups: HB (such as A-allele *vs.* C-allele: OR [95% CI] = 1.51 [1.38–1.64], *P*_(heterogeneity)_ = 0.302, *P* < 0.001; AC *vs.* CC: OR [95% CI] = 1.57 [1.38–1.79], *P*_(heterogeneity)_ = 0.156, *P* < 0.001), and PB (such as: A-allele *vs.* C-allele: OR [95% CI] = 1.31 [1.21–1.41], *P*_(heterogeneity)_ = 0.321, *P* < 0.001; AC *vs.* CC: OR [95% CI] = 1.17 [1.04–1.30], *P*_(heterogeneity)_ = 0.584, *P*=0.007) (Figures 3(a) and 3(b), Table 2).

**Figure 4:**
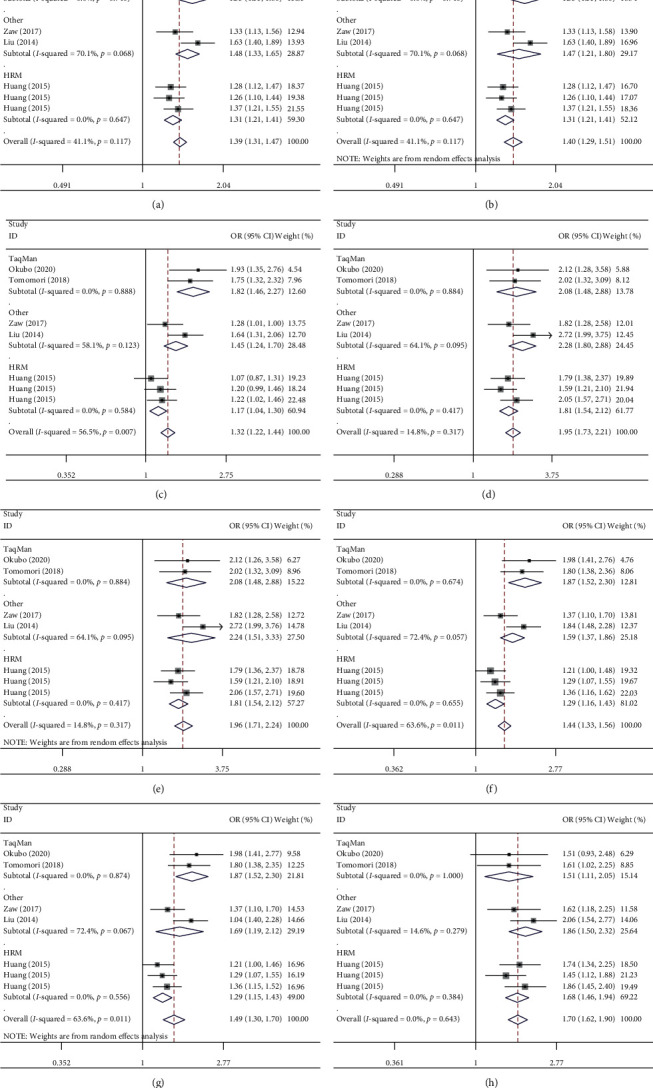
Forest plot of AF risk associated with ZFHX3 rs2106261 polymorphism in the genotype method subgroup. (a) A-allele vs. C-allele (fixed-model); (b) A-allele vs. C-allele (random-model); (c) AC vs. CC; (d) AA + AC vs. CC (fixed-model); (e) AA + AC vs. CC (random-model); (f) AA vs. CC (fixed-model); (g) AA vs. CC (random-model); and (h) AA vs. AC + CC.

To detect whether an association exists between genotype methods and the ZFHX3 rs2106261 polymorphism, we performed the next step. Several positive results were found in TaqMan (in the allelic contrast (OR = 1.55, 95% CI = 1.33–1.80, *P*=0.740 for heterogeneity, *P* < 0.001 for significance), the heterozygote comparison (OR = 1.82, 95% CI = 1.46–2.27, *P*=0.668 for heterogeneity, *P* < 0.001), AA *vs.* CC (OR = 2.06, 95% CI = 1.48–2.86, *P*_heterogeneity_ = 0.884, *P* < 0.001 for significance), the dominant model (OR [95% CI] = 1.87 [1.52–2.30], *P*_heterogeneity_ = 0.674, *P* < 0.001), and AA *vs.* AC + CC (OR [95% CI] = 1.51 [1.11–2.06], *P*_heterogeneity_ = 1.000, *P* < 0.001), high-resolution melt (in the allelic contrast (OR = 1.31, 95% CI = 1.21–1.41, *P*_heterogeneity_ = 0.647, *P* < 0.001), the heterozygote comparison (OR = 1.17, 95% CI = 1.04–1.30, *P*=0.584 for heterogeneity, *P*=0.007 for significance), AA *vs.* CC (OR = 1.81, 95% CI = 1.54–2.12, *P*_heterogeneity_ = 0.417, *P* < 0.001), the dominant model (OR = 1.29, 95% CI = 1.16–1.43, *P*=0.655 for heterogeneity, *P* < 0.001), and AA *vs.* AC + CC (OR = 1.68, 95% CI = 1.45–1.94, *P*_heterogeneity_ = 0.384, *P* < 0.001 for significance), and others (data not shown)) (Figure 4 and Table 2).

### 3.3. PRRX1 rs3903239 and AF Risk

**Figure 5:**
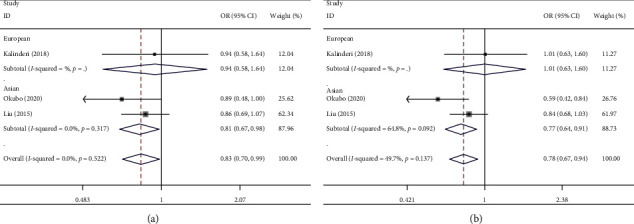
Forest plot of AF risk associated with PRRX1 rs3903239 polymorphism in the whole analysis. (a) Heterozygote comparison; (b) dominant model.

Decreased associations were found in the heterozygote comparison (OR [95% CI] = 0.83 [0.77–0.99], *P*_heterogeneity_ = 0.522, *P*=0.036, Figure 5(a) and Table 2) and dominant model (OR [95% CI] = 0.79 [0.67–0.94], *P*=0.137 for heterogeneity, *P*=0.006, Figure 5(b) and Table 2).

### 3.4. Sensitivity Analysis and Publication Bias

**Figure 6:**
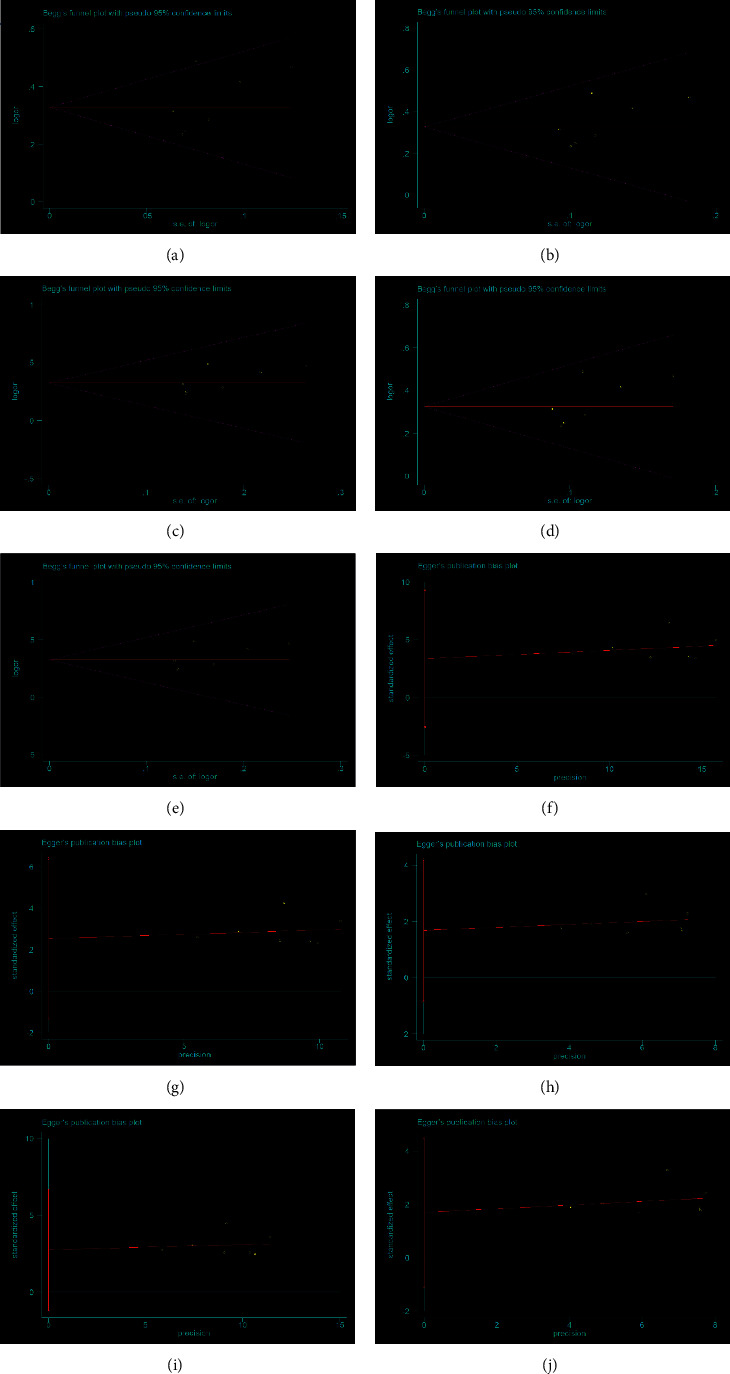
Begg's and Egger's tests for publication bias plot in all genetic models (ZFHX3 rs2106261 polymorphism). (a) A-allele vs. C-allele; (b) AC vs. CC; (c) AA vs. CC; (d) AA + AC vs. CC; (e) AA vs. AC + CC for Begg's test; (f) A-allele vs. C-allele; (g) AC vs. CC; (h) AA vs. CC; (i) AA + AC vs. CC; and (j) AA vs. AC + CC for Egger's test.

**Figure 7:**
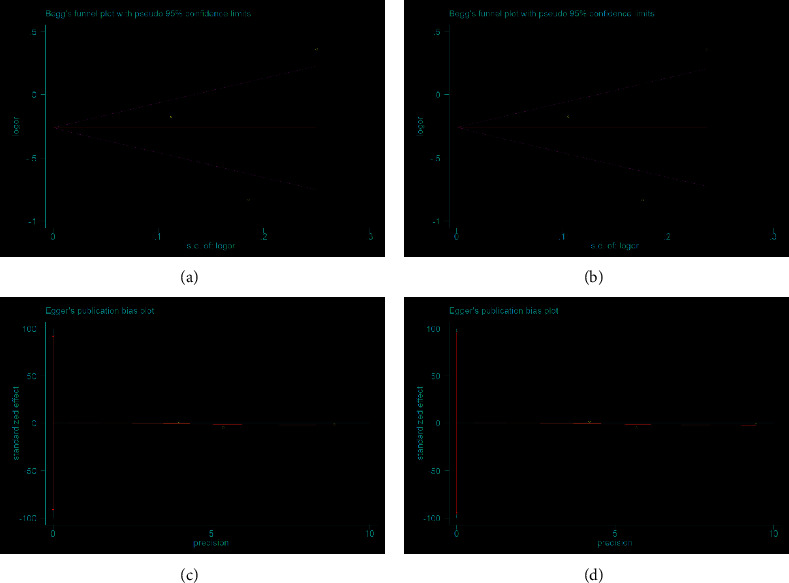
Begg's and (c, d) Egger's tests for publication bias plot in the two models (PRRX1 rs3903239 polymorphism): heterozygote comparison and dominant model.

**Figure 8:**
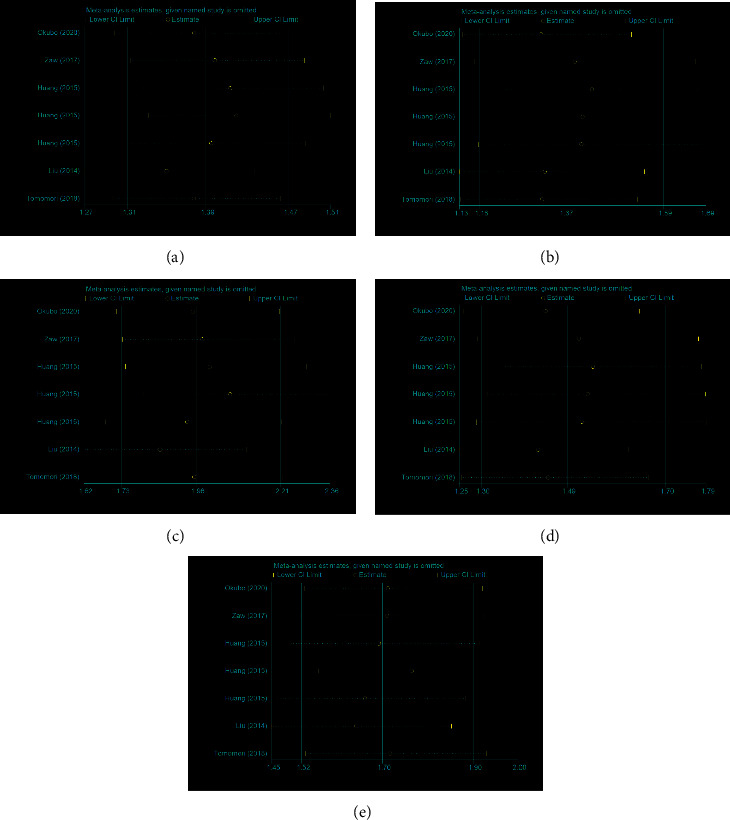
Sensitivity analysis between ZFHX3 rs2106261 polymorphism and AF risk (all five genetic models). (a) A-allele vs. C-allele; (b) AC vs. CC; (c) AA vs. CC; (d) AA + AC vs. CC; and (e) AA vs. AC + CC.

Begg's funnel chart and Egger's test were performed to assess publication bias. The results did not show any evidence of publication bias (for example, A-allele *vs.* G-allele, *t* = 1.46, *P*=0.205 (Egger's test); *z* = 1.2, *P*=0.23 (Begg's test) for ZFHX3 rs2106261, Figure 6; C-allele *vs.* T-allele, *t* = 0.11, *P*=0.933 (Egger's test); *z* = 0.0, *P*=1.00 (Begg's test) for PRRX1 rs3903239, Figure 7 and Table 3). Sensitivity analysis was performed to assess the impact of each individual study on the combined OR by removing individual studies sequentially. The results suggested that no separate study significantly affected the overall OR for ZFHX3 rs2106261 (Figure 8).

### 3.5. ZFHX3 and PRRX1 Interaction Networks

**Figure 9:**
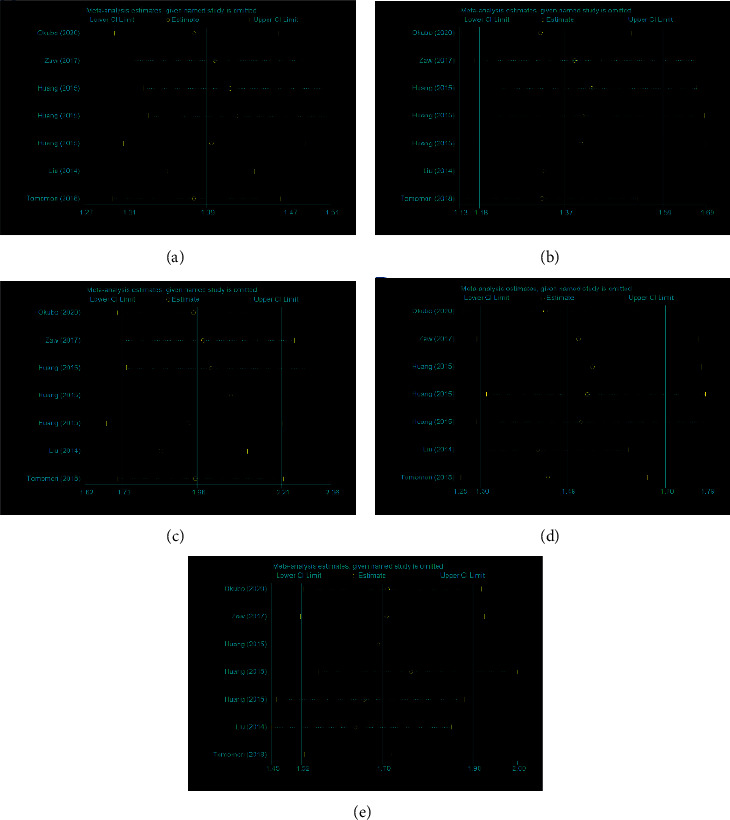
Human ZFHX3 and PRRX1 gene interactions network with other genes obtained from the String online server. At least, 10 genes have been indicated to correlate with the two abovementioned genes, respectively. (a, c) Network and ten related genes for the ZFHX3 gene; (b d) network and ten related genes for the PRRX1 gene.

A network of potential gene-gene interactions for ZFHX3 and PRRX1 genes was analyzed using the String online web page (https://string-db.org/) [33] (Figure 9). Each gene showed ten significantly related genes.

## 4. Discussion

AF is considered to be the most common supraventricular arrhythmia, affecting up to 1% of the natural population [34, 35]. With increasing age, the prevalence rate increases year by year, and the incidence of elderly cases (≥80 years) can reach 8% [36]. Many types of heart and medical diseases that increase the risk of AF include arterial hypertension, cardiomyopathies, obstructive sleep apnea, and valve dysfunction [37, 38]. In addition, based on a recent meta-analysis of GWAS for AF [11], more than 100 AF risk genetic mutations and polymorphisms have been reported, indicating that gene polymorphisms are involved in the mechanisms of AF. An increasing number of studies have shown that genetic variation may promote the pathophysiology of AF by altering protein expression and function related to various cellular activities [39].

To date, several meta-analyses of gene polymorphisms and AF susceptibility have been published and have identified associations, including chromosome 4q25 variants, CYP11B2-344T > C, and mink S38G [40–43]. A growing number of studies have identified polymorphisms in both ZFHX3 and PRRX1, and two previous meta-analyses have been involved with polymorphisms in the ZFHX3 gene, rather than the PRRX1 gene with AF susceptibility. Zhai et al. performed a meta-analysis of 10 case-control comparisons about rs7193343 polymorphism and found this polymorphism may be associated with risk of AF in the Caucasian population but not in the Asian population [44]. In addition, Jiang et al. also focused the polymorphisms for AF susceptibility through meta-analysis, and two polymorphisms in the ZFHX3 gene were analyzed (three studies about rs7193343 and only two studies about rs2106261), and no association was observed [45]. After that, other studies related to ZFHX3 gene rs2106261 polymorphism have been reported; moreover, another gene polymorphism (PRRX1 rs3903239) has been reported. Therefore, we aim to reanalyze the association between ZFHX3 rs2106261 or *PRRX1* rs3903239 polymorphism and AF risk based on previous studies.

Previously, some relative studies have been reported. Zaw et al. showed that ZFHX3 rs2106261 polymorphism was a risk marker for AF and AF-related phenotypes [27]. Huang et al. performed large-population case-control studies. They found a significant A-allelic and genotypic association with AF in three different populations [22]. In addition, more highly significant associations were observed in the combined population. Liu et al. investigated a robust association between rs2106261 and increased risk of AF (OR = 1.71, 95%CL = 1.46–2.00, *P*=1.85 × 10^−11^) [24]. However, Tomomori et al. found rs2106261 A-allele was associated with lower AF recurrence rate after pulmonary vein isolation, which was opposite to the other abovementioned studies [26]. On the other hand, Kalinderi et al. did not observe a positive association for PRRX1 rs3903239 polymorphism [18]. Okubo et al. identified five susceptible polymorphisms, including rs3903239 and rs2106261, and significant associations were demonstrated (*P*=4.2 × 10^−5^ for rs3903239 and 3.87 × 10^−6^ for rs2106261) [25]. Liu et al. confirmed that rs3903239 was a risk factor for AF (OR = 1.14, 95%CI = 1.10–1.17).

The current analysis is to evaluate the associations between ZFHX3 rs2106261 or *PRRX1* rs3903239 polymorphism and AF risk from a comprehensive analysis, involving 4719 cases and 10397 controls [24]. We found a relationship between ZFHX3 rs2106261 and AF risk; in contrast, the PRRX1 rs3903239 polymorphism functioned as a protective factor in AF development. In other words, individuals carrying the A-allele of the ZFHX3 rs2106261 polymorphism may have a high risk of AF. Individuals with the CC or CT genotype of PRRX1 might have a decreased risk for AF. These findings can help reduce the incidence of AF through early detection and possible prevention measures. Different genes or polymorphisms in the same genes may play multiple roles in the progression of AF, and this may explain the abovementioned conclusions.

In addition, the online analysis system String was applied to predict the potential functional partners of the genes, which may help to expand the range of vision of related genes. Ten genes were identified. The three highest scores of associations were for cyclin-dependent kinase inhibitor 1A (CDKN1A) (score = 0.921), runt-related transcription factor 3 (RUNX3) (score = 0.918), and transforming growth factor-beta 1 (TGF*β*1) (score = 0.900). Several studies have focused on CDKN1A and TGF*β*1, but not RUNX3, in the development of AF. Further studies should focus on the three abovementioned potentially related genes and their common polymorphisms in AF. On the contrary, the scores of related genes for PRRX1 are generally low; however, this should be verified and indicated in future research.

Although positive results were found, limitations of the current study should also be discussed. First, the literature published is relatively new, so the number of included studies is not sufficiently large based on current publications and more well-designed and larger studies in future research should be paid attention to. Second, it is possible that specific environmental and lifestyle factors influence the associations between ZFHX3 rs2106261 or PRRX1 rs3903239 polymorphism and AF including family history, age, sex, disease stage, and lifestyle. Moreover, whether the AF patients have other complications, such as hypertension, diabetes, and coronary heart disease, all the included papers have not been reported. Further comprehensive studies should include the abovementioned information. Third, there are several types of AF, such as persistent, permanent, pathologic, idiopathic, and paroxysmal. If enough data exist for different types of AF in the future, we could classify the analysis into subgroups prior to analyzing the association of the ZFHX3 rs2106261 or PRRX1 rs3903239 polymorphism with AF, which could offer more precise findings for faster translation to the clinic. Fourth, the heterogeneity was existed in our analysis, such as in total and genotype method subgroup for rs2106261 and in total for rs3903239 polymorphism. The heterogeneity for *P* value was evaluation criteria to select the model for analysis, which may result in the final results. No publication bias was found, which may reduce the influence from the heterogeneity in our analysis.

## 5. Conclusions

Our analysis illustrated that the ZFHX3 rs2106261 and PRRX1 rs3903239 polymorphisms are associated with conspicuous AF risk in Asians. Therefore, well-designed and larger studies, including information about gene-gene/gene-environment interactions, are recommended to confirm the abovementioned conclusions.

## Figures and Tables

**Table 1:** Characteristics of studies of ZFHX3 and PRRX1 genes' two common polymorphisms and atrial fibrillation risk included in our meta-analysis.

Author	Year	Country	Ethnicity	Case	Control	Case			Control			SOC	HWE	Genotype	NOS	AF type
ZFHX3 rs2106261						AA	AG	GG	AA	AG	GG					
Okubo	2020	Japan	Asian	289	287	46	143	99	32	109	146	HB	0.096	TaqMan	8	NA
Zaw	2017	Japan	Asian	411	1765	54	182	175	151	725	889	HB	0.853	Illumina	8	NA
Huang	2015	China	Asian	569	1996	99	237	233	216	869	911	PB	0.683	HRM	9	A
Huang	2015	China	Asian	641	1692	103	279	259	197	707	788	PB	0.048	HRM	9	A
Huang	2015	China	Asian	810	1627	128	369	313	149	726	752	PB	0.163	HRM	9	A
Liu	2014	China	Asian	593	996	110	299	184	99	446	451	HB	0.460	MassARRAY	8	Paroxysmal AF
Tomomori	2018	Japan	Asian	362	627	50	181	131	60	250	317	HB	0.298	TaqMan	8	Paroxysmal AF
PRRX1 rs3903239						CC	CT	TT	CC	CT	TT					
Kalinderi	2018	Greece	European	167	124	15	62	90	8	49	67	PB	0.809	RCR-RFLP	7	NA
Okubo	2020	Japan	Asian	287	287	29	139	119	59	143	85	HB	0.935	TaqMan	8	NA
Liu	2015	China	Asian	591	996	79	263	249	155	463	378	HB	0.503	MassARRAY	8	Mixed

HB: hospital based; PB: population based; SOC; source of control; PCR-RFLP: polymerase chain reaction followed by restriction fragment length polymorphism; HRM: high-resolution melt; HWE: Hardy–Weinberg equilibrium of the control group; NA: not available; NOS: Newcastle–Ottawa Scale.

**Table 2:** Stratified analyses of ZFHX3 and PRRX1 genes' two common polymorphisms on atrial fibrillation risk.

Variables	*N*	Case/Control	M-allele vs. W-allele	MW vs. WW	MM + MW vs. WW	MM vs. WW	MM vs. MW + WW
ZFHX3 rs2106261			OR(95%CI) *P*_*h*_*P I*^2^	OR(95%CI) *P*_*h*_*P I*^2^	OR(95%CI) *P*_*h*_*P I*^2^	OR(95%CI) *P*_*h*_*P I*^2^	OR(95%CI) *P*_*h*_*P I*^2^
Total	7	3674/8990	1.39(1.31–1.47)0.117 0.000 41.1%	1.37(1.18–1.59)0.007 0.000 66.5%	1.49(1.30–1.70)0.011 0.000 63.6%	1.96(1.73–2.21)0.317 0.000 14.8%	1.70(1.52–1.90)0.643 0.000 0.0%

SOC							
HB	4	1654/3675	1.51(1.38–1.64)0.302 0.000 17.7%	1.57(1.38–1.79)0.156 0.000 42.5%	1.68(1.49–1.90)0.151 0.000 43.4%	2.20(1.82–2.66)0.388 0.000 0.7%	1.73(1.45–2.07)0.520 0.000 0.0%
PB	3	2020/5315	1.31(1.21–1.41)0.321 0.000 0.0%	1.17(1.04–1.30)0.584 0.007 0.0%	1.29(1.16–1.43)0.655 0.000 0.0%	1.81(1.54–2.12)0.417 0.000 0.0%	1.68(1.45–1.94)0.384 0.000 0.0%

Genotype							
TaqMan	2	650/914	1.55(1.33–1.80) 0.740 0.000 0.0%	1.82(1.46–2.27) 0.668 0.000 0.0%	1.87(1.52–2.30) 0.674 0.000 0.0%	2.06(1.48–2.86) 0.884 0.000 0.0%	1.51(1.11–2.06) 1.000 0.000 0.0%
Other	2	1004/2761	1.47(1.21–1.80)0.068 0.000 70.1%	1.45(1.24–1.70)0.123 0.000 58.1%	1.59(1.19–2.12)0.057 0.002 72.4%	1.47(1.21–1.80)0.095 0.000 64.1%	1.86(1.50–2.32)0.279 0.000 14.5%
HRM	3	2020/5315	1.31(1.21–1.41)0.647 0.000 0.0%	1.17(1.04–1.30)0.584 0.007 0.0%	1.29(1.16–1.43)0.655 0.000 0.0%	1.81(1.54–2.12)0.417 0.000 0.0%	1.68(1.45–1.94)0.384 0.000 0.4%

PRRX1 rs3903239							
Total	3	1045/1407	0.82(0.63–1.07)0.023 0.147 73.5%	0.83(0.77–0.99)0.522 0.036 0.0%	0.79(0.67–0.94)0.137 0.006 49.7%	0.68(0.35–1.32)0.011 0.253 78.0%	0.75(0.42–1.31)0.023 0.310 73.5%

*P*_*h*_: value of the *Q*-test for the heterogeneity test; *P*: *Z*-test for the statistical significance of the OR.

**Table 3:** Publication bias tests (Begg's funnel plot and Egger's test for the publication bias test) for ZFHX3 and PRRX1 genes' two common polymorphisms (rs2106261 and rs3903239).

Egger's test						Begg's test	
Genetic type	Coefficient	Standard error	*t*	*P* value	95%CI of intercept	*z*	*P* value
ZFHX3 rs2106261							
A-allele vs. G-allele	3.372	2.313	1.46	0.205	(−2.573−9.317)	1.2	0.23
AG vs. GG	2.523	1.507	1.67	0.155	(−1.351−6.398)	1.2	0.23
AA + AG vs. GG	2.744	1.543	1.78	0.133	(−1.223−6.712)	1.2	0.23
AA vs. GG	1.671	0.977	1.71	0.148	(−0.840−4.182)	1.2	0.23
AA vs. AG + GG	1.690	1.083	1.56	0.179	(−1.094−4.475)	1.2	0.23

PRRX1 rs3903239							
C-allele vs. T-allele	1.034	9.771	0.11	0.933	(−123.117−125.186)	0.0	1.00
CT vs. TT	0.496	7.243	0.07	0.956	(−91.538−92.531)	0.0	1.00
CC + CT vs. TT	0.471	7.530	0.06	0.960	(−95.213−96.154)	0.0	1.00
CC vs. TT	0.251	3.834	0.07	0.958	(−48.468−48.971)	0.0	1.00
CC vs. CT + TT	0.290	4.031	0.07	0.954	(−50.938−51.519)	0.0	1.00

## Data Availability

The data used to support the findings of this study are available from the corresponding author upon request.
